# Cold Tolerance of *ScCBL6* Is Associated with Tonoplast Transporters and Photosynthesis in *Arabidopsis*

**DOI:** 10.3390/cimb44110378

**Published:** 2022-11-10

**Authors:** Yanli Zhou, Jingling Zhang, Changhong Zhao, Guangqiang Long, Chengli Zhou, Xudong Sun, Yunqiang Yang, Chengjun Zhang, Yongping Yang

**Affiliations:** 1 Germplasm Bank of Wild Species, Kunming Institute of Botany, Chinese Academy of Sciences, Kunming 650201, China; 2 College of Resource and Environment, Yunnan Agricultural University, Kunming 650201, China; 3 School of Information Science and Engineering, Yunnan University, Kunming 650091, China

**Keywords:** calcineurin B-like protein, cold tolerance, *Stipa capillacea*, transcriptome, tonoplast

## Abstract

Plants that are adapted to harsh environments offer enormous opportunity to understand stress responses in ecological systems. *Stipa capillacea* is widely distributed in the frigid and arid region of the Tibetan Plateau, but its signal transduction system under cold stress has not been characterized. In this study, we isolated a cDNA encoding the signal transduction protein, ScCBL6, from *S*. *capillacea*, and evaluated its role in cold tolerance by ectopically expressing it in *Arabidopsis*. Full-length *ScCBL6* encode 227 amino acids, and are clustered with CBL6 in *Stipa purpurea* and *Oryza sativa* in a phylogenetic analysis. Compared with tolerance in wild-type (WT) plants, ScCBL6-overexpressing plants (*ScCBL6-OXP*) were more tolerant to cold stress but not to drought stress, as confirmed by their high photosynthetic capacity (Fv/Fm) and survival rate under cold stress. We further compared their cold-responsive transcriptome profiles by RNA sequencing. In total, 3931 genes were differentially expressed by the introduction of *ScCBL6*. These gene products were involved in multiple processes such as the immune system, lipid catabolism, and secondary metabolism. A KEGG pathway analysis revealed that they were mainly enriched in plant hormone signal transduction and biomacromolecule metabolism. Proteins encoded by differentially expressed genes were predicted to be localized in chloroplasts, mitochondria, and vacuoles, suggesting that ScCBL6 exerts a wide range of functions. Based on its tonoplast subcellular location combined with integrated transcriptome and physiological analyses of *ScCBL6-OXP*, we inferred that ScCBL6 improves plant cold stress tolerance in *Arabidopsis* via the regulation of photosynthesis, redox status, and tonoplast metabolite transporters.

## 1. Introduction

Sessile plants are confronted with environmental stimuli that shape their geographical distribution [1,2]. In long-term organism–environment interactions, plant signaling systems work closely with functional proteins to confer stress defense. Ca^2+^ has been widely studied in cell and developmental biology owing to its constituent role and flexible oscillation within cell components [3], thereby acting as a hub for the coordination of plant growth [4]. Under natural conditions, Ca^2+^ is kept in the nanomole range in the cytoplasm and the millimolar range in cellular compartments [5,6]; in contrast, cell organelles supported by the plasma membrane, such as tonoplasts, exhibit Ca^2+^ waves upon stress initiation [7]. Ca^2+^ signaling in plant cells involves several kinds of proteins, such as calcium-dependent protein kinases (CDPKs), cell adhesion molecules (CaMs), and calcineurin B-like proteins (CBLs) [8,9]. Thus, stress signaling is closely connected with the tolerance response.

Unlike CaMs and CDPKs, which exert regulatory effects in the nucleus [10], CBLs may be anchored to the membrane via myristylation and acylation but exert a flexible regulatory effect via interaction with CBL-interacting protein kinase (CIPK). Structurally, CBLs are characterized by the presence of a conical helix–loop–helix (EF hand) motif and share sequence similarity with yeast calcineurin B subunits and animal neuronal calcium sensors [11,12,13]. The CBL family has 10 members in *Arabidopsis* and *Oryza sativa*. AtCBL1 is differentially responsive to multiple stressors, including salt, drought, and cold stress [14]. AtCBL7 modulates the low nitrate response of plants [15]. Other CBLs of *Arabidopsis* have been reported to mediate physiological responses, such as osmotic regulation, ion homeostasis [16,17], and seed development [18]. Nonetheless, the cloning and functional characterization of CBL6 and CBL8 in both model and non-model plants are still in the early stages.

Cold stress causes physiological disorders and impairs plant growth [17]. Low temperatures lead to mechanical strain on the cell wall and membrane rigidification by lamellar to hexagonal II phase transition lesions [19,20,21]. In response, plants accumulate solutes, such as malate, fumarate, and proline, to enhance freezing tolerance [22] and adjust gene expression to reprogram the cell structure and repair physiological metabolism [23]. Unraveling the cold-responsive genes and regulatory network can provide a basis for genetic engineering for crop improvement. Currently, inducer of expression (ICE)-C-repeat binding factor (CBF) is the best known cascade for cold stress tolerance [24], and other components should be investigated.

*Stipa capillacea* on the Qinghai–Tibet Plateau is tolerant to multiple abiotic stressors. Owing to its long-established domestication in the alpine climate of the Tibetan Plateau, *S. capillacea* has evolved a mechanism for cold stress tolerance. In a previous study [25], *SpCBL6* of its sibling species, *S. purpurea,* which is distributed in different regions, was found to improve cold tolerance in *Arabidopsis.* In this study, we isolated the homologous CBL6-like protein from the cold-hardy species *S. capillacea* to evaluate whether *ScCBL6* shares the same functionality and to explore the extent to which the integrated regulatory mechanisms underlying the response to cold tolerance are affected by the CBL6 protein. After ectopic expression in *Arabidopsis*, a comparative transcriptomic analysis was employed providing an in-depth understanding of the regulation of *ScCBL6* and its connection to photosynthesis.

## 2. Materials and Methods

### 2.1. Plant Preparation and Controlled Stress

*S. capillacea* seeds were collected from Qinghai–Tibet Plateau (33°26′56.5″ N, 79°48′58.4″ E). The corresponding voucher specimen was deposited at the Germplasm Bank of Kunming Institute of Botany under the ID YangYP-Q-0092. *S. capillacea* is widely distributed in the semi-arid hillsides of the alpine steppes (Figure S1). Mature and plump seeds of *S. capillacea* were surface sterilized with 2% HgCl_2_, rinsed with double-distilled H_2_O, and sowed in pots filled with nutrient soil at a density of three seeds per pot. *Arabidopsis* seeds were allowed to germinate in growth chamber at 22 °C and 60–70% humidity, then transferred to soil culture in a greenhouse at the density of three plants per pot. One-month-old seedlings were used for stress treatments, and the aboveground portion was sampled for RNA extraction.

### 2.2. Isolation and Sequence Analysis of ScCBL6

*ScCBL6* was isolated from *S. capillacea* via homology-based cloning methods, i.e., the same primers for *SpCBL6* were used to amplify the coding sequence of *ScCBL6*. Total RNA was extracted using an RNA Extraction Kit as described in Zhou et al. [25]. Forward (5′-ATGGTGGATTTCCCGGAAGG-3′) and reverse (5′-TCAAGCGTCCTCAACCTGAG-3′) primers were validated to obtain a single and sizable band. PCR was performed with Q5^®^ High-Fidelity DNA Polymerase (New England Biolabs, Ipswich, MA, USA) as follows: 94 °C for 30 s, 52 °C for 30 s, and 72 °C for 1 min for extension. The PCR products were purified and ligated to the pMD18-T vector (Takara Bio Inc., Kusatsu, Japan) and transformed into *Escherichia coli* through heat shock at 42 °C for 90 s. After selection with kanamycin, a positive clone was picked and used for Sanger sequencing. The full-length sequence was aligned with the CBL protein of several species in the family Poaceae, and the structural motifs of ScCBL6 were predicted using the web-based Motif Scan (https://myhits.isb-sib.ch/cgi-bin/motif_scan, accessed on 30 May 2017). Phylogenetic analyses, including homologous genes in *O. sativa* and *A. thaliana*, were performed using MEGA6 [26] with the maximum likelihood method.

For the overexpressing of *ScCBL6*, its coding sequence was amplified using extended forward (5′-GTCGACCCCGGGggtaccATGGTGGATTTCCCGGAAG-3′) and reverse (5′-TCAGAATTCGGATCCggtaccAGCGTCCTCAACCTGAGAG-3′) primers. The 5′ end of the primers was jointed to the plant binary vector *pRI101-GFP*, which could initiate the expression of a chimeric protein containing the target gene fused to a green fluorescent protein (GFP) sequence using a cauliflower mosaic virus (CaMV)-35S promoter. After *KpnI* digestion of the binary vector, the PCR products were subcloned into a vector, *pRI101-GFP*, through a recombinase reaction. The resulting construct was introduced into Agrobacterium tumefaciens strain GV3101, which was used to infect WT *Arabidopsis* (Columbia ecotype) via the floral dip method [27]. Transgenic seeds were screened by kanamycin resistance and positive transformants were identified using gene-specific primers. Three lines of ScCBL6-overexpressing *Arabidopsis* were used for subsequent analyses.

### 2.3. Subcellular Localization of ScCBL6

*A. tumefaciens* carrying the recombinant vector *pRI101-ScCBL6GFP* was inoculated into Luria broth and propagated for 12 h overnight. *A. tumefaciens* was introduced into a tonoplast marker-containing *Arabidopsis* via PEG-mediated protoplast transformation. *Arabidopsis* protoplasts of tender leaf were isolated as described in [28,29]. Images of green fluorescence from the GFP protein were obtained using an Olympus FV1000 laser confocal microscope (Olympus, Tokyo, Japan). The tonoplast marker, vac-rb CD3-976 [30] carrying red fluorescent protein was used to evaluate colocalization with GFP.

### 2.4. Cold and Drought Treatment of Transgenic Arabidopsis

Compared with WT *Arabidopsis* (Columbia ecotype; control), soil-cultured transgenic *Arabidopsis* was subjected to drought and cold treatment. Three plantlets were included per pot. One-month-old plants were acclimated at 4 °C for 12 h and then subjected to −6 °C for 2 h. All cold-treated plants were recovered at room temperature for another 4 d. Drought conditions were achieved by withholding water for 14 days. All treatments were replicated three times for each genotype. Quantitative reverse transcription PCR was conducted as described in [31].

### 2.5. Transcriptomic Analysis of ScCBL6-Overexpressing Transgenic Plants in Response to Cold Treatment

Samples were taken before and 4 days after cold treatment and were frozen in liquid nitrogen for RNA isolation. Quality control of total RNA was performed using a NanoDrop spectrophotometer (NanoDrop Technologies, Wilmington, DE, USA) and used for transcriptome analysis with the Illumina platform (Illumina, San Diego, CA, USA). The raw reads were filtered using Trimmomatic version 0.33 [32] (parameters: ILLUMINACLIP: TruSeq3-PE.fa:2:30:10, LEADING:3, TRAILING:3, SLIDINGWINDOW: 4:15), and clean reads were then subjected to quality control using FastQC [33]. Clean reads of each sample were pair-end assembled using TopHat 2.1.1 [34], and DEGs were identified using the Cufflinks [35] pipeline with false discovery rate (FDR) ≤ 0.05 and log2FC ≥ 2. DEGs were functionally annotated by GO and subjected to an enrichment analysis using the R package ‘clusterProfiler’. The subcellular localization of proteins encoded by DEGs was predicted using TargetP (https://omictools.com/targetp-tool, accessed on 30 March 2018). Sequence extraction and formatting were performed using TBtools [36]. All RNA-seq data were deposited in NCBI under the accession number PRJNA744953.

### 2.6. Chlorophyll Fluorescence

Chlorophyll fluorescence (Fv/Fm) for each pot was measured as described previously [37]. After adaption to the dark for 30 min, the maximum quantum yield of photosystem II for each pot was measured by exposure to a light pulse of 4000 µmol s^−1^ m^−2^ for 0.8 s. Images of pots were obtained using a chlorophyll fluorometer MAXI-Imaging Pulse-Amplitude (PAM; Walz, Effeltrich, Germany) and ImagingWin (version 2.32).

### 2.7. Water Loss Assay

To explore the water loss rate under dehydration, the leaves of 4-week-old WT and transformed *Arabidopsis* plants were cut off and allowed to dry on Whatman filter paper at 25 ± 2 °C. Net weights were recorded at 0.5, 1, 2, 4, and 6 h. The water loss rate was calculated as weight loss per unit time.

### 2.8. Antioxidative Enzyme Activity Assays

Enzymatic activity was assayed following previously described methods [38], with slight modifications. Approximately 0.1 g of fresh leaves was extracted in 50 mM Tris buffer (pH 7.0), which contained 1 mM ascorbate acid, 1 mM dithiothreitol, 1 mM glutathione, 1 mM ethylenediaminetetraacetic acid, 5 mM MgCl_2_.6H_2_O, 20% glycerol, and 1% polyvinylpolypyrrolidone. The homogenate was then centrifuged at 12,000× *g* for 6 min at 4 °C, and the supernatants were recentrifuged again under the same conditions for 16 min. Glutathione reductase (GR) activity was defined as the ability of the crude extract to reduce glutathione disulfide by the nicotinamide adenine dinucleotide phosphate auxiliary, which was quantified by absorbance at 340 nm per minute per gram fresh weight.

## 3. Results

### 3.1. Bioinformatic Analysis of ScCBL6

**Figure 1:**
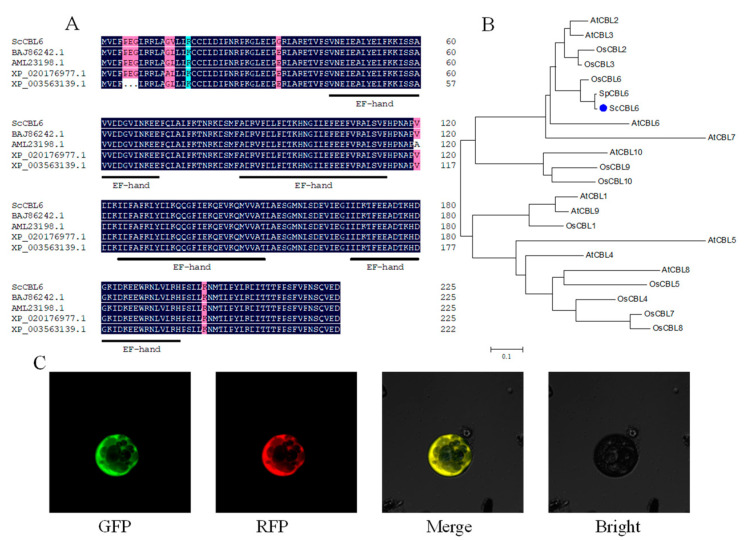
Biological properties of *ScCBL6*. (**A**) Amino acid sequence alignment of the CBL6 protein from *Stipa capillacea* (ScCBL6), *Hordeum vulgare subsp. vulgare* (BAJ86242.1), *S. purpurea* (AML23198.1), *Aegilops tauschii subsp. tauschii* (XP_020176977.1), and *Brachypodium distachyon* (XP_003563139.1). Cyan and pink indicated bases with an identity higher than 75% and 50%, respectively. (**B**) Phylogenetic analysis of ScCBL6, SpCBL6, and CBL family members in *Arabidopsis thaliana* and *Oryza sativa*. Blue dot signifies the gene of interest. (**C**) Subcellular localization of ScCBL6. Green fluorescent protein (GFP) excited at 488 nm captured using an Olympus FV1000 laser confocal microscope; red fluorescence (RFP; excited at 583 nm) indicating the tonoplast marker, which was the same as the plasmid (vac-rb CD3-976) in a previous study [30]; Merge, overlapping GFP and RFP; Bright, bright field.

*ScCBL6* was cloned from *S. capillacea* based on its homologue in *S. purpurea*. After sequencing, an open reading frame of 681 bp was designated as *ScCBL6*. The full-length gene encodes a putative protein with 226 aa residues. A protein–protein similarity search using the basic local alignment search tool against the National Center for Biotechnology Information (NCBI) database revealed that ScCBL6 shared the highest identity (98.67%) with calcineurin B-like protein 6 in *Hordeum vulgare* subsp. *vulgare* (accession no. BAJ86242.1), followed by 98.23% identity with calcineurin B-like protein in *S. purpurea* (accession no. AML23198.1), 98.23% identity with calcineurin B-like protein 6 in *Aegilops tauschii* ssp. tauschii (accession no. XP_020176977.1), and 97.35% identity with calcineurin B-like protein 6 in *Brachypodium distachyon* (accession no. XP_003563139.1) (Figure 1A). These proteins shared four classical EF hands in a Motif Scan analysis. ScCBL6 clustered with OsCBL6 in a phylogenetic analysis (Figure 1B), and the subcellular localizations of the ScCBL6 protein primarily included the tonoplast and plasma membrane (Figure 1C).

### 3.2. ScCBL6-Overexpressing Arabidopsis Demonstrated Enhanced Cold Tolerance

**Figure 2:**
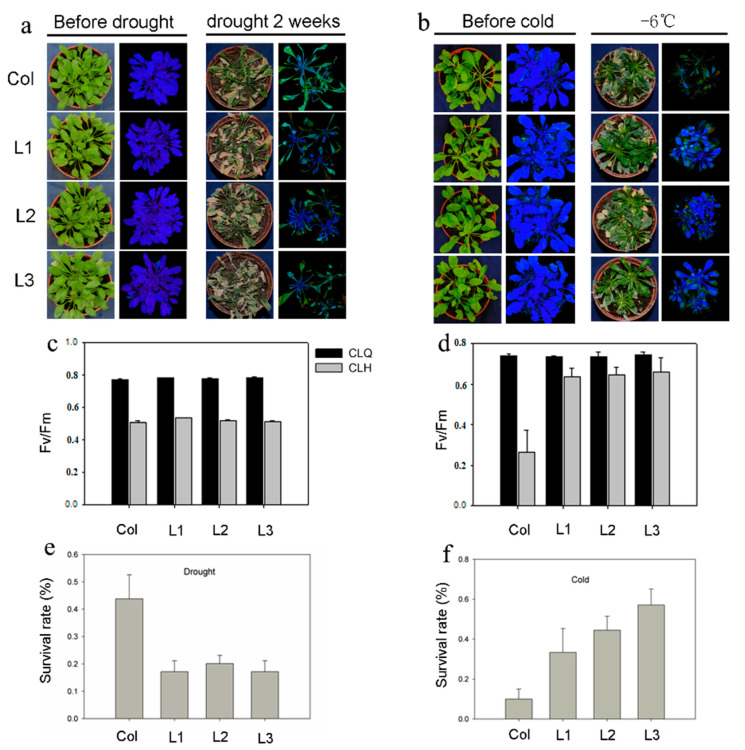
Seedling growth and survival of *ScCBL6*-overexpressing (*ScCBL6*-OXP) and wild-type (WT) plants under drought and cold stress. The upper panels (**a**,**b**) indicate the changes in the morphology and photosynthetic efficiency (Fv/Fm) after drought and cold treatment. Left-hand images (green plants) were obtained in live video mode, while right-hand images (blue plants) were observed under fluorescence light. The lower panels (**c**–**f**) show the survival rate of different transgenic lines (L1–L3) after controlled drought and cold treatments and are based on at least seven biological replicates.

**Figure 3:**
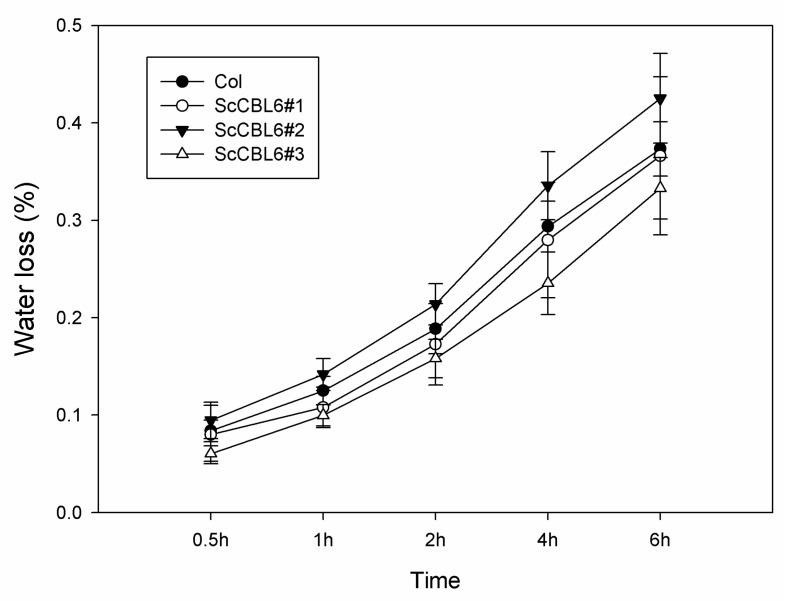
**Water loss rate of *ScCBL6*-OXP and WT leaves.** The water loss rate was calculated as the difference between weights at individual timepoints and the original weight. Col, Columbia ecotype *Arabidopsis*; numbers followed by # denote different transgenic lines of *ScCBL6*-OXP.

To explore the biological function of ScCBL6 in vivo, we generated transgenic *Arabidopsis* carrying *ScCBL6* downstream of the CaMV-35S promoter. Three independent transgenic lines were obtained by gene-specific polymerase chain reaction (PCR; Figure S2). Under natural greenhouse conditions, ScCBL6-overexpressing plants (ScCBL6-OXP) and wild-type (WT) *Arabidopsis* did not show a significant difference in growth. Similar results were obtained under drought stress, as revealed by the survival rate and Fv/Fm (Figure 2). Under freezing conditions, ScCBL6-OXP outgrew the WT in terms of survival rate and Fv/Fm. In the subsequent recovery process of cold-treated plants, the WT perished owing to regeneration loss, while ScCBL6-OXP gradually regained normal growth, indicating its higher tolerance to cold stress than that of the WT (Figure 2). The water loss rate of detached leaves did not differ significantly between ScCBL6-OXP and the WT (Figure 3).

### 3.3. Comparative Transcriptome Analysis Indicates Transcriptional Regulation of ScCBL6

**Figure 4:**
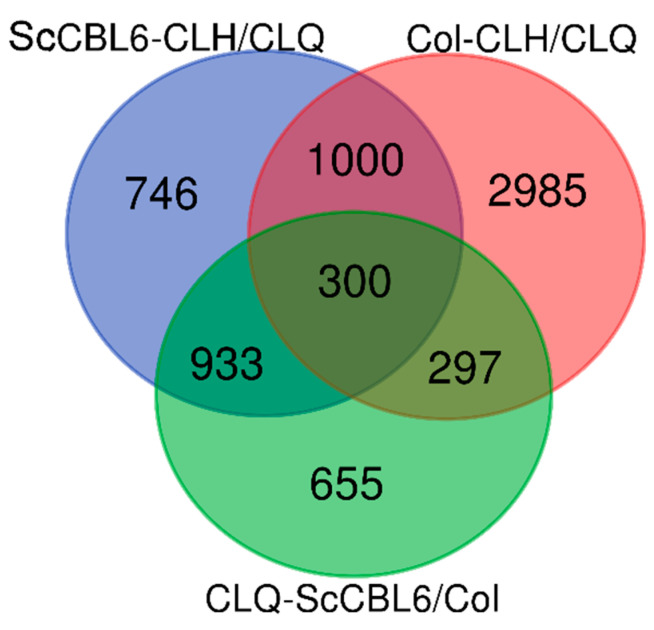
**Venn diagram of overlapping and specific differentially expressed genes (DEGs) under different conditions.** CLQ, before cold treatment; CLH, after cold treatment; ScCBL6, *ScCBL6*-overexpressing *Arabidopsis*; Col, wild-type *Arabidopsis*.

We further compared transcriptome variation between ScCBL6-OXP and WT plants under both cold and normal conditions. In total, 6916 DEGs were identified based on |log2FC| ≥ 2 and FDR ≤ 0.05, which varied between ScCBL6-OXP and WT irrespective of stress treatment. In WT *Arabidopsis*, 4582 (1000+300+297+2985) DEGs were found to be regulated by cold treatment; however, only 2185 (300+933+297+655) DEGs were modified by the overexpression of ScCBL6. In terms of cold response, ScCBL6-OXP shared 1300 (1000 + 300, account for 43.64%) overlaps with WT, while the remaining 1679 (933 + 746) showed ScCBL6 dependency (Figure 4). Among the 1300 overlapping DEGs, only 23.08% were activated without cold treatment, suggesting ScCBL6 functions in the response to cold stress.

**Figure 5:**
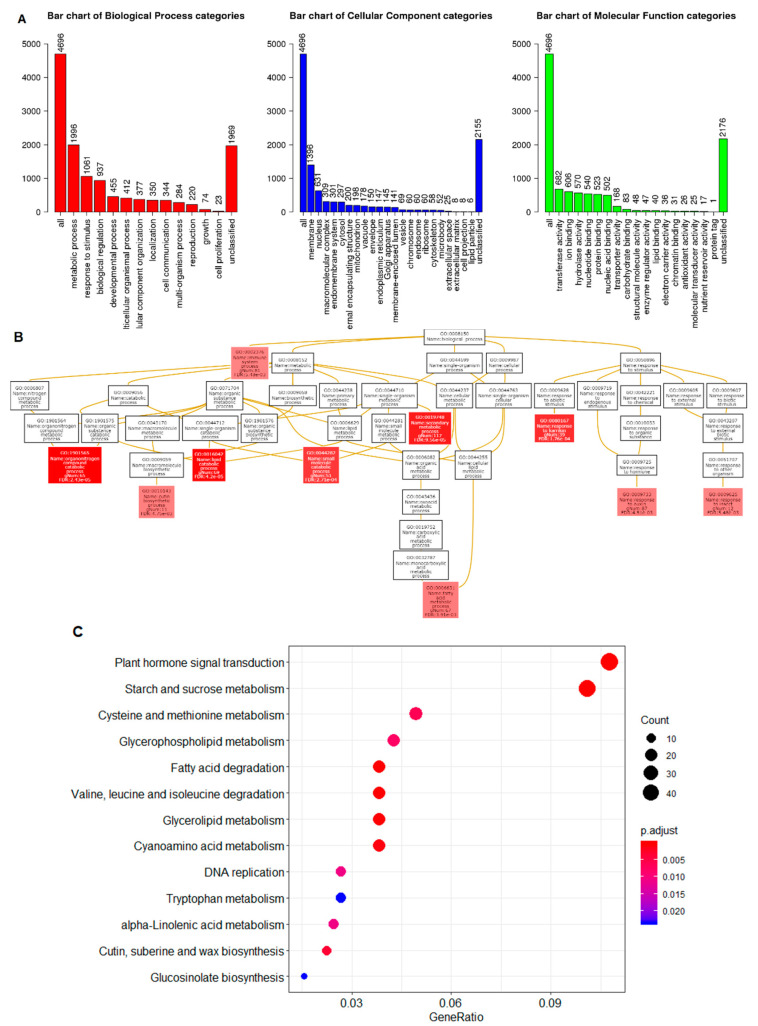
**Functional enrichment of ScCBL6-regulated DEGs**. (**A**) Summary of Gene Ontology (GO) annotation classified into three principal categories. (**B**) Directed acyclic graph of GO enrichment of DEGs. The colors in different boxes indicate significant level of FDR. (**C**) Kyoto Encyclopedia of Genes and Genomes pathway enrichment of DEGs against the *Arabidopsis* background set in clusterProfiler.

The ScCBL6-regulated DEGs were primarily enriched in the GO terms metabolic process, membranes, transferase activity in the biological process, cellular components, and molecular function categories (Figure 5A). Further GO enrichments revealed that these DEGs were primarily involved in the immune system process (GO:0002376), lipid catabolic process (GO:0016042), secondary metabolic process (GO:0019748), small molecule catabolic process (GO:0044282), organonitrogen compound catabolic process (GO:1901565), cutin biosynthetic process (GO:0010143), fatty acid metabolic process (GO:0006631), as well as the response to auxin (GO:0009733) and insects (GO:0009625) (Figure 5B). Furthermore, these mapped DEGs were primarily involved in plant hormone signal transduction, starch and sucrose metabolism, cysteine and methionine metabolism, and glycerophospholipid metabolism and fatty acid degradation, among other pathways (Figure 5C).

### 3.4. Potential Mechanism Underlying Cold Tolerance Mediated by ScCBL6

**Figure 6:**
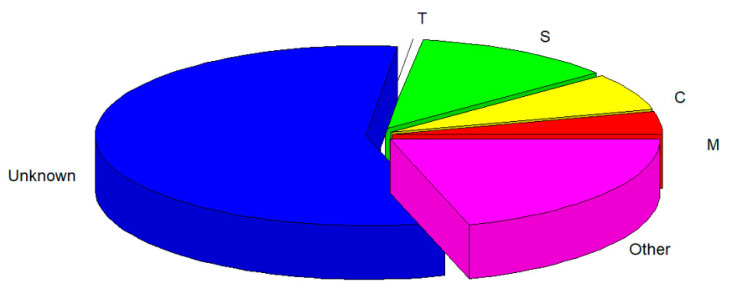
**Subcellular localization prediction of DEG expression products.** M, mitochondrion (158); S, secretory pathway (472); C, chloroplast (266); T, tonoplast (12); other (799); unknown (2226). Tonoplast-residing proteins were predicted by a BLASTx-based search of proteins identified in previous studies by Endler et al. [40] and Szponarski et al. [41], and other locations were predicted using TargetP.

**Figure 7:**
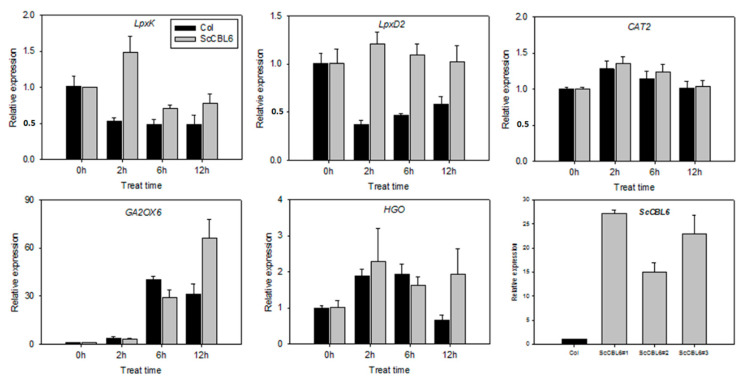
**Quantitative reverse transcription-polymerase chain reaction analysis of candidate genes regulated by ScCBL6.** The x-axis indicates the duration of cold stress; h, hour. Relative expression level calculated by the 2^−ΔΔCT^ method relative to a non-cold-treated control for each cDNA.

Based on the tonoplast localization of CBL6 in *Arabidopsis* reported by Zhang et al. [39] and the results of the subcellular localization analysis of ScCBL6 (Figure 1C), we inferred that ScCBL6 mediated cold tolerance by targeting proteins located on the membrane. Therefore, the protein sequences of ScCBL6-related DEGs were extracted and searched against a signal peptide database. The majority of the annotated DEGs were localized in chloroplasts (266) and engaged in the secretory pathway (472), and only 12 proteins were localized in the tonoplast (Figure 6). Based on the tonoplast membrane subcellular localization of ScCBL6, we focused on tonoplast proteins. Most proteins were downregulated, and only two, a polyamine choline transporter (cationic amino acid transporter 2 [CAT2]) and a carbohydrate transporter (tonoplast monosaccharide transporter1 [TMT1]), were upregulated in response to cold treatment (Table 1). Greater photosynthesis was observed in ScCBL6 overexpressing lines than in WT plants (Figure 2f). We then analyzed the interacting proteins involved in these two processes (tonoplast-related proteins and photosynthesis-related proteins) by extending their interaction network and found that 50% of CAT2 (tonoplast localized) interaction modules and 44.44% of the TMT1-interacting partners were annotated to chloroplast-related functions (Table 2). Furthermore, quantitative reverse transcription-PCR revealed that most of these genes were upregulated by ScCBL6 upon cold treatment (Figure 7), contributing to the maintenance of photosynthesis in cold-treated ScCBL6-OXP.

## 4. Discussion

Signal transduction in harsh environments is essential for the domestication of alpine plants. Ca^2+^-mediated signal transduction has been widely documented in the plant kingdom [42]. The CBL protein conveys a broad range of signals via reversible Ca^2+^ docking to its EF hand domain [43,44] and has expanded from one member in the chlorophytes (*Ostreococcus lucimarinus*) to more than 10 members in higher plants, such as *Arabidopsis* [45]. *S. capillacea* is widely distributed in the Qinghai–Tibet Plateau; however, signal transduction in the species has not yet been investigated. Based on transcriptomic data, we successfully used a homology-based method to isolate one *CBL* cDNA from *S. capillacea.* Sequence analysis revealed that the ScCBL6 protein was conserved, at least among grass types (Figure 1A). Phylogenetically, ScCBL6 showed a close relationship with CBL2 and CBL3 in both *O. sativa* and *A. thaliana*, suggesting that gene divergence preceded speciation. However, CBL2 and CBL3 are well-known vacuole proteins [18], providing evidence of the tonoplast target of ScCBL6 in *Arabidopsis*, given their high sequence similarity [39]. CBL2 and CBL3 influence seed size and embryonic development in transgenic *Arabidopsis* [18]. However, functional characterization of CBL6 has only been characterized in a few species, including *S. purpurea* and *Triticum dicoccoides* [46], providing novel albeit insufficient insights. In this study, we performed a functional analysis of ScCBL6 by ectopic expression in *Arabidopsis*, followed by a comparative transcriptome analysis of WT and transgenic lines under cold stress.

The gain-of-function mutant *ScCBL6*-OXP showed enhanced cold tolerance, but not the drought tolerance of *Arabidopsis* (Figure 2), consistent with the performance of transgenic *SpCBL6* plants [35]. *ScCBL6* shares 96.77% similarity with *SpCBL6* at the nucleotide level and a 2-aa difference at the protein level. The two sites did not functionally diverge, which is supported by the Ka/Ks result (⍵ < 1, *p* < 0.05).

**Figure 8:**
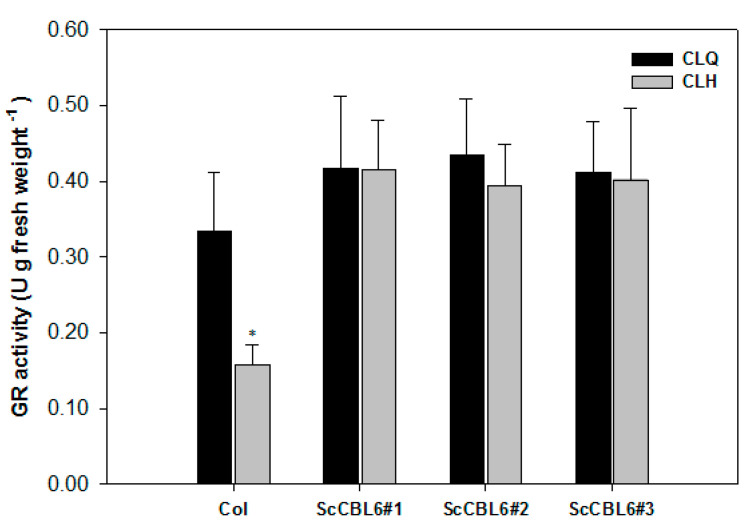
**Glutathione reductase activities of the *Arabidopsis* leaf in response to cold treatment.** Asterisk (*) shows significant difference at *p* < 0.05.

In the transcriptomic analysis, *ScCBL6* regulated the expression levels of 3931 genes (Figure 4). Genes were widely enriched in plant hormone signal transduction, secondary metabolism, and fatty acid and protein regulation (Figure 5C), indicating the various roles of ScCBL6 in plants in vivo. However, under cold stress, transcript levels of 1233 (933 + 300) of 3931 (300 + 933 + 297 + 655 + 1000 + 746) DEGs were further altered, and 746 DEGs were ScCBL6-specific cold-induced response genes, constituting the core repertoire of the cold tolerance mechanism induced by *ScCBL6*. These genes exert broad regulatory effects across tissues, such as the chloroplast, mitochondrion, and cytoplasm (Figure 6); however, only 12 genes were localized in the tonoplast. One of the two upregulated genes, CAT2, interacted with glutathione metabolism protein (OXP1) and tyrosine catabolism protein (HGO) in the STRING network (Figure S3). OXP1 catalyzes the cleavage of 5-oxo-L-proline to form L-glutamate and regulates not only the redox status, as reflected by GR activity (Figure 8), which exerts 80% of its activity in photosynthetic tissue chloroplasts [47], but also osmoregulatory substances, such as proline, and this may contribute to a low drought tolerance. Among the interacting partners of TMT1, proteins such as 1-deoxy-D-xylulose-5-phosphate synthase (CLA1) and fructokinase-like protein (FLN) are required for chloroplast development [48] and rubisco accumulation [49], consistent with the higher Fv/Fm of ScCBL6-OXP. Conversely, TMT1 is reported to increase lipid content [50], which is associated with the membrane component, in accordance with the lower ion leakage of SpCBL6-OXP, which was demonstrated in a previous study [25]. These photosynthesis and membrane-related products can impart cold tolerance [51]. The loss-of-function *tmt1* mutant reduces its transporter activity under cold stress [22,52]. Therefore, we inferred that CBL6 probably conferred cold tolerance in plants via tonoplast transporters that facilitated improved photosynthesis.

Direct interactions of ScCBL6 with cold tolerance targets were not expected because CBLs are likely to be subjected to phosphorylation by CIPKs. In this study, CIPK12, CIPK16, CIPK17, CIPK22, and CIPK25 were upregulated by cold stress in WT plants, and all these, except for CIPK12, were also upregulated in cold-treated ScCBL6-OXP. In particular, CIPK16 and CIPK25 levels increased 27.33- and 17.02-fold in cold-treated ScCBL6-OXP, respectively, indicating their potential interaction with CBL6. However, a comparison of untreated WT and ScCBL6-OXP revealed that no CIPK was significantly upregulated, and this result is consistent with the temperature dependence of CBL6/CIPK complex formation reported by Zhang et al. [39]. Thus, the cold stress response conferred by ScCBL6 involves many genes, including CAT2 and TMT1, and these are candidates for improving the cold tolerance of other crop plants. Owing to the lack of a transformation system for *S. capillacea*, further studies of the underlying mechanism and plasticity in other plants are warranted.

## 5. Conclusions

This study elucidates the complexity and polyfunctionality of the environmental signal transduction system in *S. capillacea*. In this study, *ScCBL6* isolated from *S. capillacea* induced changes throughout the cell. In *ScCBL6*-overexpressing transgenic *Arabidopsis*, cold stress tolerance was verified by a high survival rate and photosynthetic capacity. RNA sequencing revealed that metabolite transporters in tonoplasts could interact with chloroplast-related genes, affecting photosynthesis and redox regulation. ScCBL6 is a tonoplast-anchored protein, and its highly regulated functions are important to the environmental adaptation of plant vacuoles.

## Figures and Tables

**Table 1:** List of tonoplast proteins encoded by DEGs.

Transcript_ID	Gene_ID	Locus	log2FC	Description
XLOC_002346	AT1G07607	1:18517585-18521781	−1.52	Antisense long noncoding RNA
XLOC_004339	AT1G04830	1:1356083-1362019	−1.53	Ypt/Rab-GAP domain of gyp1p superfamily protein
XLOC_005225	TUB5	1:6937718-6940832	−2.13	Beta tubulin
XLOC_006801	CAT2	1:21464000-21468505	2.05	Encodes a member of the cationic amino acid transporter (CAT) subfamily
XLOC_005279	TMT1	1:7242895-7248588	2.10	Tonoplast monosaccharide transporter
XLOC_007227	AT1G65920	1:24525101-24529362	−4.39	Regulator of chromosome condensation family with FYVE zinc finger domain-containing protein
XLOC_007791	TUB1	1:28449904-28453820	−4.99	Beta tubulin
XLOC_008966	AT2G21220	2:8987583-9198383	−4.22	SAUR-like auxin-responsive protein family
XLOC_011932	TUB7	2:12642502-12646057	−2.49	Beta tubulin
XLOC_016150	AT3G62570	3:23142484-23144747	−2.08	Tetratricopeptide repeat-like superfamily protein
XLOC_019943	AT4G05275	4:4676542-4855814	−2.31	Long noncoding RNA
XLOC_023442	AT4G27270	4:13661200-13663371	−2.48	Quinone reductase family protein
XLOC_002346	AT1G07607	1:18517585-18521781	−1.52	Antisense long noncoding RNA

**Note: Transcript IDs were the assembly outputs in Cufflinks, and the corresponding gene ID and locus were obtained from reads mapping against gff files.** Log2FC values were calculated from FPKM ratio of before and after cold treated ScCBL6-OXP.

**Table 2:** GO information for CAT2 and TMT1-interacting proteins.

**CAT2-Interaction Network**			
XLOC_023086	AT4G21220	LpxD2	GO:0103118 [Name: UDP-3-*O*-(R-3-hydroxymyristoyl)-glucosamine N-acyltransferase activity]; GO:0009507 [Name: chloroplast]; GO:2001289 [Name: lipid X metabolic process]; GO:0005739 [Name: mitochondrion]
XLOC_006525	AT1G52670	BLP1	GO:0005515 [Name: protein binding]; GO:0009507 [Name: chloroplast]; GO:0006633 [Name: fatty acid biosynthetic process]
~-	AT4G05210	LpxD1	GO:0005739 [Name: mitochondrion]; GO:0009507 [Name: chloroplast]
XLOC_001368	AT1G25210	LpxC5	GO:0009507 [Name: chloroplast]
XLOC_010794	AT2G04560	LpxB	GO:2001289 [Name: lipid X metabolic process]; GO:0009245 [Name: lipid A biosynthetic process]; GO:0005543 [Name: phospholipid binding]; GO:0009507 [Name: chloroplast]; GO:0016757 [Name: transferase activity, transferring glycosyl groups]; GO:0005739 [Name: mitochondrion]
**TMT1-Interaction Network**			
XLOC_020438	AT4G15560	CLA1	GO:0019288 [Name: isopentenyl diphosphate biosynthetic process, methylerythritol 4-phosphate pathway]; GO:0005515 [Name: protein binding]; GO:0009507 [Name: chloroplast]; GO:0009570 [Name: chloroplast stroma]; GO:0008661 [Name: 1-deoxy-D-xylulose-5-phosphate synthase activity]; GO:0009228 [Name: thiamine biosynthetic process]; GO:0015995 [Name: chlorophyll biosynthetic process]; GO:0016114 [Name: terpenoid biosynthetic process]; GO:0046872 [Name: metal ion binding]; GO:0016744 [Name: transferase activity, transferring aldehyde or ketonic groups]; GO:0052865 [Name: 1-deoxy-D-xylulose 5-phosphate biosynthetic process]
XLOC_007437	AT1G69200	FLN2	GO:0019200 [Name: carbohydrate kinase activity]; GO:0009658 [Name: chloroplast organization]; GO:0042644 [Name: chloroplast nucleoid]; GO:0042793 [Name: transcription from plastid promoter]; GO:0005737 [Name: cytoplasm]; GO:0009662 [Name: etioplast organization]
XLOC_015664	AT3G54090	FLN1	GO:0042644 [Name: chloroplast nucleoid]; GO:0005515 [Name: protein binding]; GO:0005634 [Name: nucleus]; GO:0005737 [Name: cytoplasm]; GO:0016773 [Name: phosphotransferase activity, alcohol group as acceptor]; GO:0043621 [Name: protein self-association]; GO:0009658 [Name: chloroplast organization]; GO:0019200 [Name: carbohydrate kinase activity]; GO:0042793 [Name: transcription from plastid promoter]
XLOC_000080	AT1G02400	GA2OX6	GO:0009639 [Name: response to red or far red light]; GO:0052635 [Name: C-20 gibberellin 2-beta-dioxygenase activity]; GO:0052634 [Name: C-19 gibberellin 2-beta-dioxygenase activity]; GO:0009416 [Name: response to light stimulus]; GO:0009507 [Name: chloroplast]; GO:0009686 [Name: gibberellin biosynthetic process]; GO:0045487 [Name: gibberellin catabolic process]; GO:0051213 [Name: dioxygenase activity]; GO:0005737 [Name: cytoplasm]; GO:0046872 [Name: metal ion binding]; GO:0055114 [Name: oxidation-reduction process]
XLOC_022652	AT4G14150	KIN12A	GO:0000914 [Name: phragmoplast assembly]; GO:0007018 [Name: microtubule-based movement]; GO:0007112 [Name: male meiosis cytokinesis]; GO:0055046 [Name: microgametogenesis]; GO:0008017 [Name: microtubule binding]; GO:0005737 [Name: cytoplasm]; GO:0016887 [Name: ATPase activity]; GO:0008574 [Name: ATP-dependent microtubule motor activity, plus-end-directed]; GO:0003777 [Name: microtubule motor activity]; GO:0005524 [Name: ATP binding]; GO:0005874 [Name: microtubule]; GO:0009524 [Name: phragmoplast]; GO:0005515 [Name: protein binding]; GO:0080175 [Name: phragmoplast microtubule organization]; GO:0005871 [Name: kinesin complex]; GO:0009507 [Name: chloroplast]

## Data Availability

Raw RNA-sequencing data were deposited in NCBI under the accession number PRJNA744953.

## Supplementary Materials

The following supporting information can be downloaded at: https://www.mdpi.com/article/10.3390/cimb44110378/s1, Figure S1: Highland distribution of *Stipa capillacea*; Figure S2: Gene-specific PCR identification of transgenic plants; Figure S3: Protein–protein interaction networks of CAT2 and TMT1.
