# Supplementary material for: Cold Tolerance of ScCBL6 Is Associated with Tonoplast Transporters and Photosynthesis in Arabidopsis
Source: Curr Issues Mol Biol. 2022 Nov 10;44(11):5579–92. doi: 10.3390/cimb44110378 (PMC9689059; doi:10.3390/cimb44110378)
Supplement: Supplementary file 1 [file cimb-44-00378-s001.zip › cimb-1964923-supplementary.pdf]

Additional files

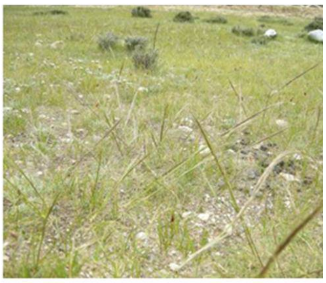

Figure S1. Highland distribution of *Stipa capillacea.*

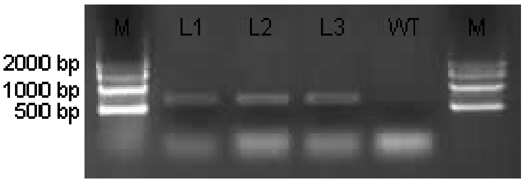

Figure S2. Gene-specific PCR identification of transgenic plants

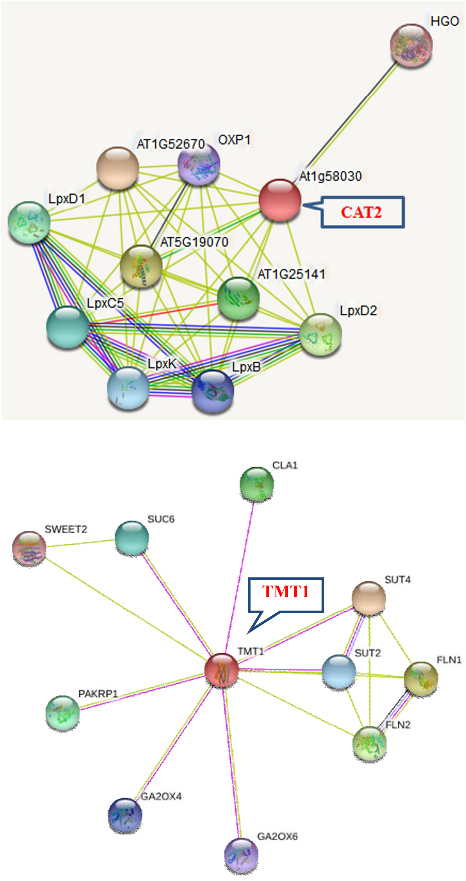

Figure S3. Protein interaction networks of CAT2 and TMT1
